# Efficient Tissue Culture Protocol for *Magnolia lucida* (Magnoliaceae) and Confirmation of Genetic Stability of the Regenerated Plants

**DOI:** 10.3390/plants9080997

**Published:** 2020-08-05

**Authors:** Lu Kang, Keyuan Zheng, Yuqing Xie, Yanwen Deng, Yina Yu, Mulan Zhu, Ruchun Xi, Xiaomei Deng

**Affiliations:** 1 Guangdong Key Laboratory for Innovative Development and Utilization of Forest Plant Germplasm, Guangzhou 510642, China; gnaklu@163.com (L.K.); serumia@163.com (Y.X.); hinmentang@gmail.com (Y.D.); gzyyn@163.com (Y.Y.); 2 College of Forestry and Landscape Architecture, South China Agricultural University, Guangzhou 510642, China; 3 Shanghai Chenshan Plant Science Research Center, Chinese Academy of Sciences, Shanghai 201602, China; 13086036031@163.com (K.Z.); mlzhu@cemps.ac.cn (M.Z.)

**Keywords:** sterilization, shoot initiation, shoot proliferation, rooting, genetic fidelity assessment

## Abstract

*Magnolia lucida* (Magnoliaceae) is classified as an endangered species by the International Union for Conservation of Nature. It has high commercial value owing to its attractive tree shape and flowers. We adopted an excellent genotype of *M. lucida* as the parent material and established a mini-cut orchard through grafting to provide trunk shoots explants over the long-term. Optimal sterilization was achieved using a combination of 75% ethanol for 30 s, one percent benzalkonium bromide for five minutes, and 0.1% mercuric chloride for five minutes. Modified Murashige and Skoog medium (ML) was the optimal medium for the growth of *M. lucida*. Addition of one mg/L of 6-benzyl adenine (BA) and 0.05 mg/L of α-naphthaleneacetic acid (NAA) to the medium increased the shoot induction rate to 95.56%, and the ML medium containing 0.4 mg/L BA and 0.04 mg/L NAA achieved the maximum multiplication rate (284.56%). Dark treatment for seven days, followed by continuous light treatment could better resolve the challenge of difficult rooting in *M. lucida* plants. Using random amplified polymorphic DNA and inter simple sequence repeat markers, we confirmed the genetic uniformity and stability of the regenerated plants. Our protocol should be helpful for the propagation and conservation of this endangered plant.

## 1. Introduction

**Figure 1:**
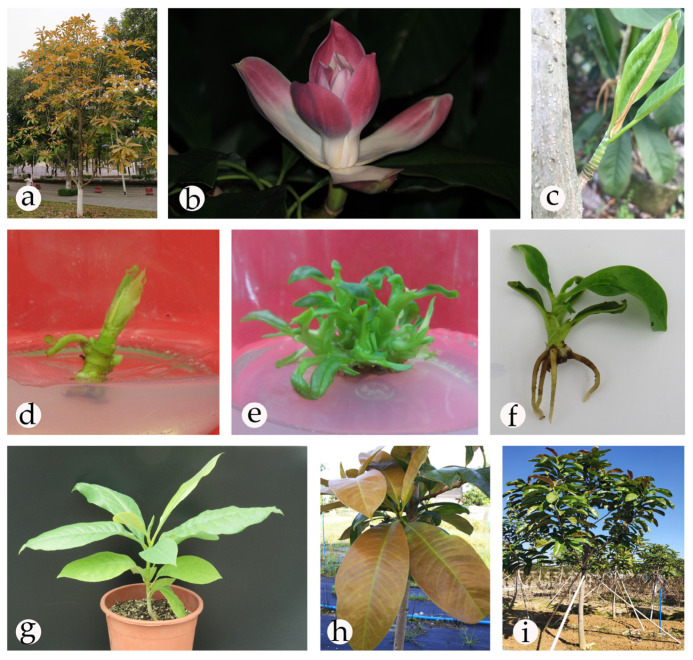
Micropropagation of *Magnolia lucida* using trunk shoot explants; (**a**) mother plant (excellent genotype); (**b**) flower; (**c**) trunk shoot explant; (**d**) shoot initiation for 20 days; (**e**) multiple shoot regeneration for 20 days; (**f**) root of regenerated plantlets; (**g**) the plantlets after acclimatization for 90 days; (**h**) regenerated plantlets with ochre brown tender leaves; (**i**) regenerated plantlets in the field.

*Magnolia lucida* (B. L. Chen & S. C. Yang) V. S. Kumar, which belongs to Magnoliaceae, is a popular ornamental tree that can be used for landscaping [1]. The tepals of *M. lucida* are obovate-oblong, with purplish-red middle and upper parts, and a white base. The leaves of this excellent genotype are highly ornamental; the tender leaves are ochre brown and mature leaves are dark green (Figure 1a,b) [2]. Additionally, *M. lucida*, with a straight trunk and an excellent material structure, is a valued timber species in Southeast Yunnan, China [1,3]. However, owing to the destruction of its habitat, overdevelopment, and low natural regeneration [4,5], *M. lucida* is classified as an endangered species by the International Union for Conservation of Nature [6]. Thus, there is an urgent need to protect and utilize *M. lucida* using effective methods.

Currently, the propagation of *M. lucida* is usually done by generating seedlings and grafting. However, the progeny propagated by seed have wide variation, and the excellent characteristics of the mother plant cannot be preserved well in them [7,8]. In addition, the occurrence of a partial crown is common in grafting and cutting propagation, which reduces the ornamental value. Moreover, propagation by grafting cannot produce enough progeny because the cuttings of the mother plant are limited. With its effectiveness, plant tissue culture has made significant contributions to the addressing of reproduction-related challenges in Magnoliaceae [9]. Several precious *Magnolia* species, such as *M. punduana*, *M. sirindhorniae*, and *M. dealbata*, have been successfully propagated using tissue culture [10,11,12]. However, owing to the high levels of phenols in magnolias, it is challenging to establish an aseptic system; additionally, it is difficult to induce rooting in the regenerated plants [9]. Thus, the in vitro propagation of most endangered Magnoliaceae such as *M. lucida* has not been accomplished [3]. The difficulty in the in vitro propagation of *M. lucida* is due to the limited explant availability because the twigs of a mature tree or withes at the base of the trunk are very thick and thus not conducive to explant sterilization and shoot induction. Therefore, the selection of a suitable explant is paramount for the successful micropropagation of *M. lucida*.

Several factors, such as medium composition and growth conditions, may cause considerable variations in the regenerated plants in the process of tissue culture [13,14]. Therefore, the evaluation of genetic uniformity of regenerated plants is particularly important. Random amplified polymorphic DNA (RAPD) and inter simple sequence repeat (ISSR) markers are extensively used to assess genetic fidelity, and they have been successfully used in many plants, such as *Vinca minor*, *Foeniculum vulgare*, and *Pisum sativum* [15,16,17]. 

The objective of this study was to establish a reliable method for conserving and reproducing *M. lucida* excellent genotypes by tissue regeneration, and to analyze the genetic stability of regenerated plants.

## 2. Results

### 2.1. Establishment of an Aseptic System

In this study, sterilization effects of three chemical disinfectants for *M. lucida* explants were compared alone or in combination (Table 1). Among the seven treatments, the best result was achieved with the 75% ethanol (C_2_H_6_O) for 30 s, 1% benzalkonium bromide (C_21_H_38_BrN) for 5 min, and 0.1% mercuric chloride (HgCl_2_) for 5 min combination. With this combination, the survival rate of explants increased up to 78%. The lowest survival rate was observed with benzalkonium bromide alone, followed by ethanol. The survival rate of explants increased, and then decreased with the extension of mercuric chloride disinfection; when 0.1% mercuric chloride was used for 7 min, the survival rate was reduced and the explants showed a rotted appearance. Other treatments generally induced a moderate response in the explants.

### 2.2. Shoot Initiation

The explants were induced on four media to select the optimal one (Table 2). Among the four media, ML (a modified MS medium) was the optimal medium for shoot induction, followed by Douglas-fir cotyledon revised (DCR) medium [18]. Although shoot induction and growth were observed on Murashige and Skoog (MS) medium [19] and woody plant medium (WPM) [20], the shoots that appeared were unhealthy. Furthermore, the shoots had a low induction rate and grew slowly, and then stopped growing or died in the last stage. The shoot showed faster elongation on ML medium, appearing verdant green and robust (Figure 1d). In addition, we found that the induction effect of α-naphthaleneacetic acid (NAA) was superior to that of indole-3-butyric acid (IBA) at the similar concentrations. Based on the rate of induction and growth, the optimal medium for the initial induction was ML + 6-benzyladenine (BA) (1 mg/L) + NAA (0.05 mg/L).

### 2.3. Shoot Proliferation

In the subsequent experiments, 16 combinations of BA and NAA concentrations were compared to optimize growth (Table 3). The optimal BA concentration was 0.4 mg/L and the optimal NAA concentration was 0.04 mg/L. Among the various combinations tested, the highest number (5.3) of shoots per explant was obtained on ML medium containing 0.6 mg/L BA and 0.04 mg/L NAA. The maximum shoot multiplication rate (284.56%) was obtained on ML medium containing 0.4 mg/L BA and 0.04 mg/L NAA, on which the shoot clusters were verdant green and thriving, without defoliation or hyperhydricity (Figure 1e).

### 2.4. Rooting and Acclimatization

Without dark treatment, only a few shoots could regenerate roots and the rooting effects were not favorable (Table 4), with flavescent and withered leaves observed. In contrast, after 7 d of dark treatment, the status of the plants improved significantly. In addition, the rooting rate improved significantly (*p* ≤ 0.05) following the use of a combination of NAA and IBA, whereas medium supplemented with NAA alone presented considerably lower rooting rates. Following dark treatment, the maximum percentage of rooting (87.78%) and the highest average root number of 4.89 were observed on ML medium supplemented with 0.6 mg/L NAA and 1 mg/L IBA. The roots were healthy, and the leaves were verdant green and thriving (Figure 1f).

The survival rate of the plantlets transferred to plastic cups reached 89% after 90 d, and their leaves were verdant green (Figure 1g). The lignified plants transferred to the field showed good growth. The regenerated plants were phenotypically identical to their mother plant, with ochre brown tender leaves and dark green mature leaves (Figure 1h,i).

### 2.5. Genetic Fidelity Assessment

**Figure 2:**
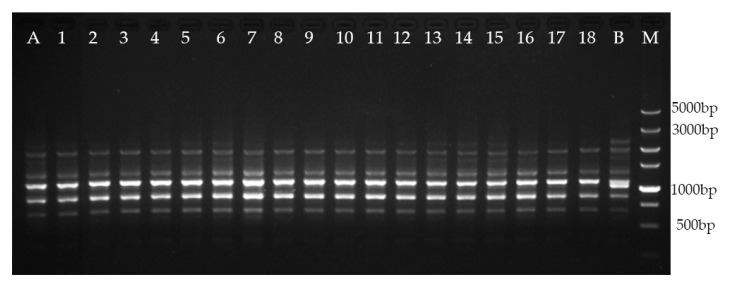
Amplification patterns generated with ISSR primer UBC811. Lane M: molecular marker (100–5000 bp); lane A: mother plant; lanes 1–18: regenerated plants from trunk shoot explant; lane B: negative control.

**Figure 3:**
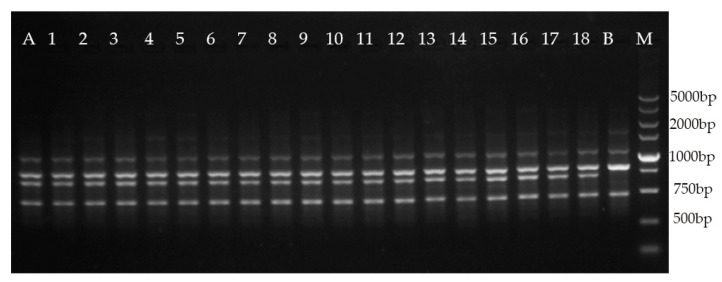
Amplification patterns generated with RAPD primer S31. Lane M: molecular marker (100–5000 bp); lane A: mother plant; lanes 1–18: regenerated plants from trunk shoot explant; lane B: negative control.

In this study, 16 RAPD primers generated 70 distinct and scorable bands (Table 5), which ranged from 250 to 3000 bp, and the number of scorable bands generated with single RAPD primers varied from three to six. Six ISSR primers generated 31 distinct and scorable bands, which ranged from 500 to 3000 bp. The number of scorable bands generated with single ISSR primers ranged from four to five. No polymorphic bands were discovered between the regenerated plants and the mother plant, compared with those in the negative control (Figure 2; Figure 3). The results show the genetic consistency between the regenerated plants and the mother plant.

## 3. Discussion

The selection of appropriate explant types is essential for plant tissue culture [21]. In the preliminary experiment, we used the twigs of a mature tree and the withes at the base of the trunk as explants. However, we failed to establish an efficient sterile system using the method, as the explants were very thick, difficult to sterilize, and displayed severe browning. The trunk bud sprouts from a latent bud on the trunk, and latent buds on the trunk of *M. lucida* are relatively easy to germinate. In the present study, we used 3–5-cm-long trunk shoots as explants to overcome the above-mentioned challenges. This may be because the trunk buds are relatively thin and exposed to the environment for a short time, thereby increasing the disinfection efficiency.

To establish plant tissue culture, explant sterilization is necessary, which is especially difficult when dealing with endangered species, with limited explant availability [22,23]. Therefore, effective disinfection treatments, without damaging explant tissue, are important for the initiation in *M. lucida* [24]. This study indicated that a combination of 75% ethanol (applied for 30 s), 1% benzalkonium bromide (applied for 5 min), and 0.1% mercuric chloride (applied for 5 min) was the most suitable sterilization treatment of *M. lucida* explants. Prior to sterilization with mercuric chloride, 75% ethanol and 1% benzalkonium bromide could partially dissolve the epicuticular wax, and thus increase the effectiveness of subsequent disinfection [25]. A previous study indicated that mercuric chloride is more effective in combination with other chemical disinfectants than alone [26]. We also found that long-term sterilization (7 min) with mercuric chloride was toxic to explants, initially causing necrosis and then killing the explants, which is consistent with previous findings in *Jatropha curcas* [27].

The basal medium is important for micropropagation, and different plants require different nutritional components [28,29]. Among the four basal media tested in this study, ML medium better supported the regeneration and proliferation of shoots, which were poor on MS medium and WPM. This may be explained by the higher concentrations of NO_3_^-^ and NH_4_^+^ in MS medium than in ML medium, as excessive NH_4_^+^ and NO_3_^-^ have negative effects on organogenesis, such as hyperhydricity [30,31]. Meanwhile, the shoot performance was also poor on WPM and DCR media because of the low NH_4_^+^ and NO_3_^-^ concentrations hinders the growth of shoots [32]. Thus, it can be concluded that both high and low concentrations of these ions were not conducive for shoots growth and proliferation of *M. lucida*. In addition, explants grow better in ML medium and may benefit from moderate concentrations of Ca^2+^ because calcium is essential for the formation of the cell wall and facilitates cell elongation. When the Ca^2+^ concentrations are insufficient, cell wall synthesis is hindered, in turn adversely affecting cell division, causing stunted explant growth, and increasing hyperhydricity rate [33,34].

The basal medium can only guarantee the survival of the culture and minimal physiological activities. The plant can initiate cell division, morphogenesis, organ differentiation, and development only when the medium is supplemented with appropriate plant growth regulators [35]. Auxins and cytokinins are generally considered the most important growth regulations in in vitro propagation [36]. A balance between auxins and cytokinins is necessary for the formation of buds and root [35]. It has been reported that an optimal combination of BA and NAA in a culture medium could significantly enhance shoot proliferation [37]. Thus, during organogenesis of *Echinacea pallida* from leaf explants, the most optimal combination was 6 mg/L BA and 0.02 mol/L NAA [38], whereas Mamun et al. [39] achieved the maximum shoot multiplication rate of *Albizia lebbeck* on MS medium supplemented with 2.5 mg/L BA and 0.2 mg/L NAA. However, in our study, ML medium supplemented with 0.4 mg/L BA and 0.04 mg/L NAA was the most suitable combination for shoot proliferation and elongation. This result was different from that in other Magnoliaceae members, which showed the maximum multiplication rate under the combination of 0.2 mg/L BA and 0.01 mg/L NAA [11]. The differences may be due to the highly varying requisite concentration of each type of regulator, depending on the cultured plant and cultural conditions [40]. 

It has been reported that plants belonging to Magnoliaceae have poor root formation [41]. In this study, we found that dark treatment could effectively increase the rooting rate of *M. lucida*, because appropriate dark treatment can increase endogenous phenol levels and increase the utilization of carbohydrates, which in turn leads to a higher rooting percentage [42,43]. The result is consistent with those of studies performed on *Parakmeria lotungensis* and *Sinomanglietia glauca* [44]. A combination of 0.6 mg/L NAA and 1 mg/L IBA and 7 d of initial dark treatment could effectively overcome the poor rooting by *M. lucida*. This result is different from that for *Magnolia sirindhorniae*, which can achieve a rooting rate of 95.67% only through the combination of appropriate concentrations of auxin [11]. The positive effects of a dark environment on different species need to be further explored.

Many factors can influence the stability of plants during tissue culture [45]. Therefore, it is important to evaluate the genetic stability of regenerated plants [46,47,48]. In the present study, RAPD and ISSR markers were used to analyze the genetic stability of regenerated plants after two years of subculture. No polymorphic bands were observed between the regenerated plants and the mother plant, when compared with those in the negative control, which confirmed the genetic uniformity of the regenerated plants. Our results suggest that the direct induction of multiple shoots could minimize the likelihood of instability, consistent with the results of previous studies [11].

The growth conditions play a significant role in optimizing and regulating the growth of in vitro plants. In these experiments, we directly placed the culture in a low-light and low-temperature environment to better control browning. Dark treatment was used to increase the rooting rate and good results were obtained, but further research on the influence of pH, medium sugar content, and other conditions is needed.

## 4. Materials and Methods

### 4.1. Plant Materials and Sterilization

We used the excellent genotypes of *M. lucida* as the parent material and established a miniature cutting orchard by grafting to provide long-term sampling. The mother parent, with an excellent genotype, came from South China Agricultural University (113°19′ E, 23°04′ N) (Figure 1). Trunk shoots were used as explants. Prior to the collection of explants, the mother plant was sprayed with carbendazim every 3 d. When the trunk shoots were 3–5 cm long, they were cut and brought to the laboratory. After being thoroughly washed with a 5% (*v*/*v*) liquid detergent solution, the shoots were rinsed under running tap water for 2 h. Subsequently, based on the research of Cui et al. [11] and Deng [44], we set up seven different sterilization schemes, with 100 explants each (Table 1). Thereafter, in the initial induction of explants, the sterilized explants were planted vertically on MS supplemented with 1 mg/L BA and 0.05 mg/L NAA (determined in a preliminary experiment). Following 20 d of culture, survival rates were recorded.

### 4.2. Media and Culture Conditions

MS, WPM, DCR, and ML media were used in this study. The ML basal medium was obtained by continuously improving the macronutrient in MS medium according to the growth status of the explants in preliminary experiments. The ML basal medium comprised the following macronutrient components (mg/L): NH_4_NO_3_, 600; K_2_SO_4_, 660; KH_2_PO_4_, 200; MgSO_4_·7H_2_O, 370; and Ca(NO3)_2_, 556. The other nutrients and vitamins were similar to those in the MS medium.

For shoot induction and proliferation, 30 g/L sucrose was added to the medium and 15 g/L sucrose to the rooting medium [8,10]. The pH of the media was adjusted to 5.8; then, the media was solidified with agar (6 g/L) [11] and autoclaved at 121 °С for 18 min. To prevent browning, the inoculated culture was placed in a low light and low temperature environment (24 °С ± 2 °С, fluorescent light 12-h/day photoperiod, 20 μmol m^−2^s^−1^).

### 4.3. Shoot Initiation

Aseptic explants were cultured in four shoot induction media (Table 2). Each medium was supplemented with 1 mg/L BA, and 0.05 mg/L NAA or IBA to achieve the maximum rate of shoot initiation. There were 12 treatments in total, and the treatments were repeated three times, with each repeat consisting of 30 explants. After 20 d of incubation, the induction rate and the growth state were recorded.

### 4.4. Shoot Proliferation

The ML medium was also selected as the basal medium to compare the combination effects of different BA and NAA concentrations on shoot proliferation (Table 3). BA was tested at concentrations of 0.2, 0.4, 0.6, and 0.8 mg/L, and NAA was tested at concentrations of 0.02, 0.04, 0.06, and 0.08 mg/L. The experiment was repeated three times, with each repeat consisting of 30 explants. After 20 d of incubation, the multiplication rate, number of shoots per explant, and growth state were recorded.

### 4.5. Rooting

Robust shoots (≥1.2 cm in height) were harvested and transferred to ML medium supplemented with different concentrations of NAA (0.3, 0.6, 0.9 mg/L) and IBA (0, 0.5, 1 mg/L) (Table 4). Each treatment was set in triplicate, with 30 explants and two groups. The first group was immediately cultured under light conditions and the second group was incubated for 7 d in dark (the tissue culture seedlings were placed in a dark environment) and then transferred to light conditions. After 30 d of culture, the rooting rate and average root number were recorded.

### 4.6. Acclimatization

After 30 d, 300 plants with well-developed roots (root length > 1 cm, robust) were transferred to a greenhouse for approximately 7–10 d. Thereafter, the plantlets were removed from the culture vessels and thoroughly washed under running tap water to remove adhering media. Subsequently, the plantlets were transplanted to plastic cups containing a mixture of peat soil, perlite, and coconut bran at a ratio of 3:1:1 (*v*/*v*/*v*); then, the seedlings were watered and covered with a shading net and provided timely ventilation. The percentage of survival was recorded after 90 d.

### 4.7. Genetic Fidelity Assessment

After two years of subculture of buds, the genetic stability was determined. Genomic DNA was extracted from young leaves of 18 regenerated plants and their mother plant using the cetyltrimethylammonium bromide method [49]. In addition, the genomic DNA of another *M. lucida* plant developed from a seed was extracted as the negative control. All plant DNA samples were analyzed using 16 RAPD and six ISSR primers (Table 5), which were selected for the genetic analysis of Magnoliaceae in previous studies [50,51]. The primers were provided by Tsingke Biological Technology (Beijing, China) and were used according to the previous studies and initial experiments. RAPD and ISSR DNA amplification was performed in 25 μL of a reaction mixture containing 1.0 μL of template DNA (20 ng/μL), 12.5 μL of 2× Taq Plus Master Mix (Beijing ComWin Biotech Co., Ltd., Beijing, China), 1.0 μL of primer (10 μM), and 10.5 μL of ddH_2_O. The ISSR amplification was performed under the following conditions: initial DNA denaturation at 94 °С for 5 min, followed by 35 cycles of denaturation at 94 °С for 45 s, annealing at 58 °С for 1 min, and extension at 72 °С for 2 min, with a final extension at 72 °С for 10 min. The RAPD amplification was performed under the following conditions: initial DNA denaturation at 94 °С for 5 min, followed by 40 cycles of denaturation at 94 °С for 1 min, annealing at 40 °С for 1 min, and extension at 72 °С for 2 min, with a final extension at 72 °С for 10 min.

The ISSR and RAPD amplification products were subjected to electrophoresis on 1.5% agarose gels in 1.5% Tris–acetate–EDTA buffer using a 5000-bp DNA marker (Takara, Kyoto, Japan), and the gels were stained with 0.25 μg/mL ethidium bromide. A gel documentation system (Bio-Rad, Hercules, CA, USA) was used for visualization, and the analysis using the RAPD and ISSR primers was repeated three times.

### 4.8. Statistical Analysis

The following formulae were used to calculate different plant regeneration parameters:

- Induction rate (%) = number of induced explants/total number of explants × 100
- Multiplication rate (%) = total number of shoots ≥ 0.3 cm/number of initial shoots on subcultured explants × 100
- Number of shoots per explant = total number of shoots ≥ 0.5 cm/number of explants
- Rooting rate (%) = number of rooted explants/number of explants × 100
- Average root number = total number of roots/number of rooted explants

IBM SPSS Statistics v23 (Armonk, NY, USA) was used for statistical analyses. Data were subjected to analysis of variance (ANOVA). Significant differences among means were calculated using Duncan’s multiple range test at *p* ≤ 0.05. The results are presented as mean ± standard error of three replicates.

## 5. Conclusions

We established an efficient and reliable regeneration protocol for micropropagation of an endangered plant, from trunk shoot explants of an excellent genotype of *M. lucida*. The regenerated plants, which were propagated using this protocol, showed good growth and had verdant green and thriving leaves, as well as well-developed roots. The use of RAPD and ISSR genetic markers confirmed the genetic uniformity of the regenerated plants. These results indicated that the direct induction of multiple shoots could safely be used as an efficient tissue culture method for propagation of *M. lucida*. To the best of our knowledge, this is the first report of genetically sustainable *M. lucida* tissue culture from trunk shoot explants.

## Figures and Tables

**Table 1:** Effectiveness of different sterilization schemes.

No.	Treatment Composition (Duration)			Survival (%)
	Ethanol 75% (s)	Benzalkonium Bromide 1% (min)	Mercuric Chloride 0.1% (min)	
1	-	5	-	4
2	-	-	5	36
3	30	-	-	27
4	30	5	1	56
5	30	5	3	68
6	30	5	5	78
7	30	5	7	61

**Table 2:** Effects of different media on the initial shoot induction.

No.	Medium	BA (mg/L)	NAA (mg/L)	IBA (mg/L)	Induction Rate (%) (Mean ± SE; *n* = 3)	Observation
1	ML	1	0.05	-	95.56 ± 3.14 ^a^	Robust and green shoots
2	ML	1	-	0.05	85.56 ± 1.57 ^b^	Robust and green shoots
3	ML	1	-	-	60.00 ± 2.72 ^de^	Slow growth of shoots
4	DCR	1	0.05	-	74.44 ± 4.16 ^c^	Green shoots
5	DCR	1	-	0.05	63.33 ± 2.72 ^d^	Slow growth of shoots
6	DCR	1	-	-	48.89 ± 3.14 ^ghi^	Slow growth of shoots
7	WPM	1	0.05	-	57.78 ± 4.16 ^def^	Slow growth of shoots
8	WPM	1	-	0.05	51.11 ± 5.67 ^fgh^	Slow growth of shoots
9	WPM	1	-	-	42.22 ± 1.57 ^i^	Slow growth of shoots
10	MS	1	0.05	-	53.33 ± 5.44 ^efg^	Hyperhydricity
11	MS	1	-	0.05	43.33 ± 4.71 ^hi^	Hyperhydricity
12	MS	1	-	-	33.33 ± 5.44 ^j^	Hyperhydricity, browning

Different superscript letters in the same column indicate significant differences at *p* ≤ 0.05. *n* = 3 indicates three replicates. BA: 6-benzyladenine; NAA: α-naphthaleneacetic acid; IBA: indole-3-butyric acid; ML: modified MS medium; DCR: Douglas-fir cotyledon revised medium; WPM: Woody plant medium; MS: Murashige and Skoog medium.

**Table 3:** Effects of different combinations of BA and NAA concentrations on shoots proliferation.

No.	BA (mg/L)	NAA (mg/L)	Multiplication Rate (%) (Mean ± SE, *n* = 3)	Number of Shoots per Explant (≥ 0.5 cm) (Mean ± SE, *n* = 3)	Growth State of Buds
1	0	0	98.87 ± 4.93 ^l^	1.20 ± 0.08 ^h^	Shorter shoots
2	0.2	0.02	247.85 ± 5.65 ^ef^	4.07 ± 0.21 ^d^	Shorter shoots
3	0.2	0.04	253.54 ± 3.27 ^de^	4.60 ± 0.14 ^b^	Shorter shoots
4	0.2	0.06	235.91 ± 3.18 ^g^	3.63 ± 0.12 ^e^	Shorter shoots
5	0.2	0.08	215.90 ± 6.27 ^hi^	3.00 ± 0.08 ^f^	Partial callus
6	0.4	0.02	274.69 ± 3.15 ^b^	4.70 ± 0.08 ^b^	Robust shoots
7	0.4	0.04	284.56 ± 3.88 ^a^	5.17 ± 0.12 ^a^	Robust shoots
8	0.4	0.06	267.85 ± 5.82 ^bc^	4.50 ± 0.22 ^b^	Robust shoots
9	0.4	0.08	250.69 ± 4.52 ^def^	4.43 ± 0.21 ^bc^	Partial shoots
10	0.6	0.02	259.58 ± 8.67 ^cd^	4.37 ± 0.21 ^bcd^	Robust shoots
11	0.6	0.04	268.81 ± 2.76 ^bc^	5.30 ± 0.08 ^a^	Robust shoots
12	0.6	0.06	251.68 ± 8.26 ^def^	4.40 ± 0.22 ^bcd^	Robust shoots
13	0.6	0.08	241.84 ± 4.12 ^fg^	4.10 ± 0.29 ^cd^	Slightly crinkled leaf, callus
14	0.8	0.02	208.79 ± 2.54 ^ij^	3.67 ± 0.09 ^e^	Crinkled leaf
15	0.8	0.04	224.23 ± 3.70 ^h^	3.30 ± 0.08 ^f^	Crinkled leaf
16	0.8	0.06	204.14 ± 2.12 ^j^	3.27 ± 0.17 ^f^	Flavescence, partial callus
17	0.8	0.08	177.42 ± 2.40 ^k^	2.57 ± 0.05 ^g^	Flavescence, callus

Different superscript letters in the same column indicate significant differences at *p* ≤ 0.05. *n* = 3 indicates three replicates.

**Table 4:** Effects of different combinations of NAA and IBA concentrations on rooting.

Group	No.	NAA (mg/L)	IBA (mg/L)	Percentage of Rooting (%) (Mean ± SE, *n* = 3)	Average Root Number (Mean ± SE, *n* = 3)
Directly cultured by light	1	0.3	0	0 ^j^	0 ^i^
	2	0.3	0.5	28.89 ± 3.14 ^gh^	1.34 ± 0.07 ^gh^
	3	0.3	1	36.67 ± 7.20 ^fg^	1.53 ± 0.07 ^efg^
	4	0.6	0	0 ^j^	0 ^i^
	5	0.6	0.5	41.11 ± 6.85 ^def^	1.81 ± 0.20 ^e^
	6	0.6	1	52.22 ± 5.67 ^cd^	2.59 ± 0.14 ^d^
	7	0.9	0	13.33 ± 2.72 ^i^	1.15 ± 0.11 ^h^
	8	0.9	0.5	27.78 ± 4.16 ^gh^	1.74 ± 0.20 ^ef^
	9	0.9	1	20.00 ± 2.72 ^hi^	1.44 ± 0.14 ^fgh^
Darkness for 7 days	1	0.3	0	27.78 ± 4.16 ^gh^	1.78 ± 0.13 ^e^
	2	0.3	0.5	51.11 ± 6.85 ^cd^	2.59 ± 0.15 ^d^
	3	0.3	1	60.00 ± 7.20 ^c^	3.35 ± 0.17 ^c^
	4	0.6	0	37.78 ± 4.16 ^efg^	2.34 ± 0.11 ^d^
	5	0.6	0.5	71.11 ± 6.29 ^b^	4.26 ± 0.27 ^b^
	6	0.6	1	87.78 ± 4.16 ^a^	4.89 ± 0.13 ^a^
	7	0.9	0	42.22 ± 6.85 ^def^	1.52 ± 0.23 ^efg^
	8	0.9	0.5	54.44 ± 8.31 ^c^	3.40 ± 0.30 ^c^
	9	0.9	1	48.89 ± 5.67 ^cde^	2.24 ± 0.14 ^d^

Different superscript letters in the same column indicate significant differences at *p* ≤ 0.05. *n* = 3 indicates three replicates.

**Table 5:** Random amplified polymorphic DNA (RAPD) and inter simple sequence repeat (ISSR) primers, and the number and size of the amplified fragments.

Primer Code	Sequence (5′–3′)	No. of Scorable Bands	Range of Amplification (bp)
RAPD			
S10	CTGCTGGGAC	6	750–3000
S11	GTAGACCCGT	5	1000–3000
S17	AGGGAACGAG	3	500–3000
S18	CCACAGCAGT	5	750–3000
S22	TGCCGAGCTG	5	750–3000
S31	CAATCGCCGT	4	250–2000
S38	CTGGGGCTGA	5	750–3000
S40	GTTGCGATCC	4	1000–3000
S69	CTCACCGTCC	5	1000–3000
S144	GTGACATGCC	5	750–3000
S154	TGCGGCTGAG	4	750–2000
S155	ACGCACAACC	4	1000–3000
S160	AACGGTGACC	4	750–2000
S163	GGACTGCAGA	5	2000–3000
S173	CTGGGGCTGA	3	750–2000
S174	CTGGGGCTGA	3	1000–2000
Total		70	
ISSR			
UBC811	(GA)_8_G	5	500–3000
UBC835	(SG)_8_YC	4	750–3000
UBC840	(GA)_8_YT	5	500–3000
UBC842	(GA)_8_YG	6	750–3000
UBC844	(CA)_8_RG	5	1000–3000
UBC864	(ATG)_6_	6	750–5000
Total		31	
